# Upscaling between an agent-based model (smoothed particle approach) and a continuum-based model for skin contractions

**DOI:** 10.1007/s00285-022-01770-y

**Published:** 2022-09-03

**Authors:** Q. Peng, F. J. Vermolen

**Affiliations:** 1 Mathematical Institute, Leiden University, Niels Bohrweg 1, 2333 CA Leiden, The Netherlands; 2 Delft Institute of Applied Mathematics, Delft University of Technology, Mekelweg 4, 2628 CD Delft, The Netherlands; 3 Computational Mathematics Group, Discipline Group Mathematics and statistics, Faculty of Science, Hasselt University, Campus Diepenbeek, Agoralaan Gebouw D, 3590 BE Diepenbeek, Belgium

**Keywords:** Traction forces, Mechanics, Smoothed particle approach, Agent-based modelling, Finite-element methods, Continuum-based modelling, 92-10, 92-08, 35Qxx, 65Mxx, 65Zxx

## Abstract

Skin contraction is an important biophysical process that takes place during and after recovery of deep tissue injury. This process is mainly caused by fibroblasts (skin cells) and myofibroblasts (differentiated fibroblasts which exert larger pulling forces and produce larger amounts of collagen) that both exert pulling forces on the surrounding extracellular matrix (ECM). Modelling is done in multiple scales: agent-based modelling on the microscale and continuum-based modelling on the macroscale. In this manuscript we present some results from our study of the connection between these scales. For the one-dimensional case, we managed to rigorously establish the link between the two modelling approaches for both closed-form solutions and finite-element approximations. For the multi-dimensional case, we computationally evidence the connection between the agent-based and continuum-based modelling approaches.

## Introduction

Wound healing is a spontaneous process for the skin to cure itself after an injury. It is a complicated combination of various cellular processes that contribute to resurfacing, reconstituting and restoring of the tensile strength of the injured skin. For superficial wounds where only the epidermis is damaged, they heal without any issues. However, for severe injuries, in particular dermal wounds, they may result in various pathological problems, such as contractures.

Contractures concur with disabilities and disfunctioning of joints of patients, which cause a significantly severe impact on patients’ daily life. Contractures are recognized as excessive and problematic contractions, which occur due to the pulling forces exerted by the (myo)fibroblasts on their direct environment, i.e. the extra cellular matrix (ECM). Contractions mainly start occurring in the proliferation phase of wound healing: the fibroblasts are migrating towards the wound and differentiating into myofibroblasts due to the high concentration of transforming growth factor-beta (TGF-beta). Usually, $5-10\%$ reduction of wound area has been observed in clinical trials. A more detailed biological description can be found in Enoch and Leaper (2008); Cumming et al. (2009); Haertel et al. (2014); Martin (1997).

In our previous work (Peng and Vermolen 2020b), a formalism to describe the mechanism of the displacement of the ECM has been used, which is firstly developed by Boon et al. (2016) and improved further by Koppenol (2017). To model skin contraction, which results from the cellular traction forces applied on the ECM, the momentum balance equation (i.e. the elasticity equation) is combined with the immersed boundary approach (Ferziger et al. 2002). Regarding the elasticity equation with point forces, we realized that the solution to the partial differential equation is singular in the sense that the formal solution does not reside in the same function space as the finite element solution does for two- and higher dimensional problems. Hence, we developed various alternatives to improve the accuracy of the solution in Peng and Vermolen (2020c, 2022).

We have been working with agent-based models so far, which model the cells as individuals and define the formalism of pulling forces by superposition theory. However, once the wound scale is larger, the agent-based model is increasingly expensive from a computational perspective, and hence, the cell density model, which is a continuum-based model, is preferred. In this manuscript, we investigate and discover the connections between these two models, in the perspective of modelling the mechanism of pulling forces exerted by the (myo)fibroblasts. Since the consistency between the smoothed particle approach (SP approach) and the immersed boundary approach has been proven both analytically and numerically (Peng and Vermolen 2019a, b), we select the SP approach here due to its continuity and smoothness, to compare with the cell density model using finite-element methods.

The objective of this paper is to demonstrate the consistency between the smoothed particle approach and the cell density approach, therefore, the same physical scale is considered in the current study. The cell density approach, which amounts to the continuum scale is mainly considered in scales that are in the order millimeters or larger. For such scales, the number of cells becomes too large and hence it is no longer computationally feasible to use individual cells that have sizes that range in the order of micrometers or centimeters and hence a continuum-based approach with cell densities is necessary so that cellular processes such as migration (random walk, chemotaxis etc.), cell division/proliferation, cell death can be dealt with by the incorporation of several terms in partial differential equations. For small scales, ranging in magnitudes that are smaller than millimeters, averaged cell densities no longer make sense and hence the continuum-based models become less useful and hence partial differential equations can no longer approximate the dynamics of cellular processes. Another reason for the use of agent-based models is their formulation in terms of measurable quantities such as cell forces, cell migration velocities etc. For even smaller scales that range within nanometers, the partial differential equations that we employ for the balance of momentum is no longer applicable. Then one has to use molecular dynamics-based simulations for all processes, including the mechanics.

The manuscript is structured as follows. We start introducing both models in one dimension in Sect. 2, then in Sect. 3 we extend the models to two dimensions. Section 4 displays the numerical results in one and two dimensions. Finally, some conclusions are shown in Sect. 5.

## Mathematical models in one dimension

This section considers one-dimensional solutions for two models: the smoothed particle model and the cell density model. Furthermore, convergence of the analytic solutions of these approaches, as well as convergence of the numerical solution are considered in this chapter. The treatment of the one-dimensional case is relatively straightforward due to the simple nature of the exact solutions.

The balance of momentum, is modelled by linear elasticity based on isotropy. In the Navier-Cauchy equation, inertia is neglected. For a general dimensionality, the Navier-Cauchy equation consists of Eqs. (16), (17) and (18) that are displayed in Sect. 3. We consider the one-dimensional version of the Navier-Cauchy equation in an isotropic and continuous domain, hence, the equations are given by

$$\begin{aligned} -\frac{d\sigma }{dx}= & {} f, \quad \text{ Equation } \text{ of } \text{ Equilibrium, }\\ \epsilon= & {} \frac{du}{dx}, \quad \text{ Strain-Displacement } \text{ Relation, }\\ \sigma= & {} E\epsilon , \quad \text{ Constitutive } \text{ Equation. } \end{aligned}$$

By substituting $E=1$, the equations above can be combined to Poisson equation in one dimension:

1 $$\begin{aligned} -\frac{d^2u}{dx^2}=f. \end{aligned}$$

### Smoothed particle approach

In Peng and Vermolen (2019b), a smoothed particle (SP) approach is developed as an alternative of the Dirac Delta distribution describing the point forces exerted by the biological cells, in the application of wound healing. By specifying the force expression *f* in Eq. (1) and considering $N_s$ cells, the smoothed particle approach (Peng and Vermolen 2020a, c, 2021) is given by

2 $$\begin{aligned} (BVP_{SP})\left\{ \begin{aligned} -\frac{d^2u}{dx^2}&=P_{SP}\sum _{i=1}^{N_s}\delta '_{\varepsilon }(x-s_i), x\in (0,L),\\ u(0)&=u(L)=0, \end{aligned} \right. \end{aligned}$$

where $P_{SP}$ is the magnitude of the forces, $\delta _{\varepsilon }(x)$ is the Gaussian distribution with variance $\varepsilon$ and $s_i$ is the centre position of biological cell *i*. The boundary conditions close the problem so that it admits a uniquely defined solution. One can solve the partial differential equations (PDEs) with finite-element methods. The corresponding weak form is given by

$$\begin{aligned} (WF_{\mathrm{SP}})\left\{ \begin{aligned}&\text {Find }u\in H_0^1((0, L))\text {, such that}\\&\int _0^L u'\phi 'dx=\int _0^L\sum _{i=1}^{N_s}P_{SP}\delta '_{\varepsilon }(x-s_i)\phi dx,\\&\text {for all }\phi \in H_0^1((0, L)). \end{aligned} \right. \end{aligned}$$

The existence and uniqueness of the $H^1_0$-solution follows as well from the application of the Lax–Milgram theorem (Braess 2007), where it is immediately obvious that the bilinear form in the left-hand side is symmetric and positive definite.

### Cell density approach

A cell density approach is often used in the large scale, so that the computational efficiency is much improved compared with the agent-based model. According to the model in Koppenol (2017), the force in two dimensions is proportional to the divergence of $n_c\cdot {\varvec{I}}$, where $n_c$ is the local density of the biological cells and ${\varvec{I}}$ is the identity tensor. In one dimension, this becomes $f = P_{den}\frac{dn_c}{dx}$, the cell density approach is expressed as:

3 $$\begin{aligned} (BVP_{den})\left\{ \begin{aligned} -\frac{d^2u}{dx^2}&=P_{den}\frac{dn_c}{dx}, x\in (0,L),\\ u(0)&=u(L)=0, \end{aligned} \right. \end{aligned}$$

where $P_{den}$ is the magnitude of the forces. The corresponding weak form is given by

$$\begin{aligned} (WF_{\mathrm{den}})\left\{ \begin{aligned}&\text {Find }u\in H_0^1((0, L))\text {, such that}\\&\int _0^L u'\phi 'dx=\int _0^L P_{den}n'_s\phi dx,\\&\text {for all }\phi \in H_0^1((0, L)). \end{aligned} \right. \end{aligned}$$

### Consistency between two models

**Fig. 1:**
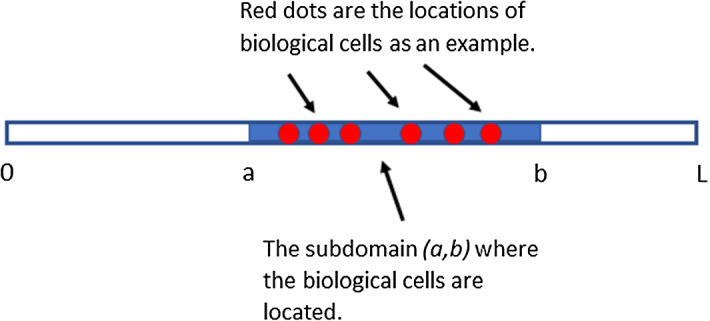
A schematic representation of the computational domain (0, *L*) in one dimension. The subdomain $(a,b)\subset (0,L)$ with $0<a<b<L$ where the biological cells (red dots in the figure) are *only* located is marked as a blue bar

In the application of wound healing, we assume an artificial wound embedded within the computational domain. Therefore, for the one-dimensional case, we define the computational domain as (0, *L*) as aforementioned, and biological cells are located in the subdomain $(a, b)\subset (0, L)$ where $0<a<b<L$; see Fig. 1 for a schematic representation.

#### Analytical solutions with specific locations of biological cells

To express the analytical solution, it is necessary to determine the locations of the biological cells, such that the cell density can be written as an analytical function of the positions. We assume that there are $N_s$ cells distributed uniformly in the subdomain (*a*, *b*) of the computational domain (0, *L*). Hence, the distance between the center positions of any two adjacent biological cells is constant, which we denote by $\varDelta s = (b-a)/N_s$ and the first and the $N_s$-th cell are located at $x=a+\varDelta s/2$ and $x=b-\varDelta s/2$, respectively. With homogeneous Dirichlet boundary conditions, and suppose $P_{SP} = P\varDelta s$ and variance $\varepsilon = \varDelta s$, the boundary value problem of the SP approach is expressed as

4 $$\begin{aligned} (BVP^1_{SP})\left\{ \begin{aligned} -\frac{d^2u_1}{dx^2}&=P\varDelta s\sum _{i=1}^{N_s}\delta '_{\varDelta s}(x-s_i), x\in (0,L),\\ u_1(0)&=u_1(L)=0, \end{aligned} \right. \end{aligned}$$

where *P* is a positive constant and $s_i$ is the centre position of the biological cells. Utilizing the superposition principle, the analytical solution (i.e. the displacement at arbitrary position of the domain) is given by

5 $$\begin{aligned} u_1(x) = P\varDelta s\sum _{i=1}^{N_s}\frac{1}{2}\left\{ \left( \frac{x}{L}-1\right) {{\,\mathrm{erf}\,}}\left( \frac{s_i}{\sqrt{2}\varDelta s}\right) +\frac{x}{L}{{\,\mathrm{erf}\,}}\left( \frac{L-s_i}{\sqrt{2}\varDelta s}\right) -{{\,\mathrm{erf}\,}}\left( \frac{x-s_i}{\sqrt{2}\varDelta s}\right) \right\} , \end{aligned}$$

where ${{\,\mathrm{erf}\,}}(x)$ is the error function defined as ${{\,\mathrm{erf}\,}}(x)=\frac{2}{\sqrt{\pi }}\int _{0}^{x}\exp (-t^2)dt$ (Weisstein 2010). Note that the solution satisfies the Dirichlet boundary conditions in $(BVP_{SP}^1)$.

Since the biological cells are uniformly located between *a* and *b* ($0<a<b<L$), $\frac{dn_c}{dx}$ can be rephrased as

$$\begin{aligned} \frac{dn_c}{dx} = \left\{ \begin{aligned}&\frac{1}{t},&a-\frac{t}{2}< x< a+\frac{t}{2},\\&-\frac{1}{t},&b-\frac{t}{2}<x<b+\frac{t}{2},\\&0,&\text{ otherwise, } \end{aligned} \right. \end{aligned}$$

where *t* is a small positive constant. In other words, $n_c(x)$ increases linearly in $(a-t/2,a+t/2)$ and decreases linearly in $(b-t/2,b+t/2)$ — with respect to *x* — and stays constant elsewhere 1/*t* in $(a+t/2,b-t/2)$ and zero for $x \in (0,a-t/2) \cup (b+t/2,L)$. Taking *t* to zero, the above expression converges to $n_c(x) = \delta (x-a)-\delta (x-b)$. Hence, the boundary value problem of the cell density model can be written as

6 $$\begin{aligned} (BVP^1_{den})\left\{ \begin{aligned} -\frac{d^2u_2}{dx^2}&=P\frac{dn_c}{dx}\rightarrow P(\delta (x-a)-\delta (x-b)), x\in (0,L),\\ u_2(0)&=u_2(L)=0, \end{aligned} \right. \end{aligned}$$

where $\delta (x)$ is the Dirac Delta distribution and *a* and *b* are the left and right endpoint of the subdomain (where biological cells are uniformly located) respectively. The analytical solution (i.e. the displacement at arbitrary position of the domain) is then expressed as

7 $$\begin{aligned} u_2(x) = P(G(x,a) - G(x,b)), \end{aligned}$$

where $G(x, x')$ is the Green’s function (Haberman 1983), defined by

$$\begin{aligned}G(x, x') = (1-\frac{x'}{L})x-max(x-x', 0),\end{aligned}$$

in the computational domain (0, *L*). Note that the solution satisfies the Dirichlet boundary conditions in $(BVP_{den}^1)$.

We will demonstrate the convergence between $u_1(x)$ and $u_2(x)$ as $\varDelta s\rightarrow 0^+$. First, we introduce Chebyshev’s Inequality:

##### Lemma 1

(Chebyshev’s Inequality (Olkin and Pratt 1958)) Denote *X* as a random variable with finite mean $\mu$ and finite variance $\sigma ^2$. Then for any positive $k\in {\mathbb {R}}$, the following inequality holds:

$$\begin{aligned} {\mathbb {P}}(|X-\mu |\geqslant k)\leqslant \frac{\sigma ^2}{k^2}, \end{aligned}$$

where ${\mathbb {P}}(A)$ is the probability of event *A*. The above inequality can also be rephrased as

$$\begin{aligned} {\mathbb {P}}(|X-\mu |\leqslant k)\geqslant 1-\frac{\sigma ^2}{k^2}. \end{aligned}$$

The proof of Chebyshev’s Inequality is standard (Olkin and Pratt 1958), and therefore we do not give it here.

##### Proposition 1

Let $u_1(x)$ as described in Eq. (4) be the exact solution to $(BVP^1_{SP})$ and $u_2(x)$ as described in Eq. (6) be the exact solution to $(BVP_{den}^1)$. As $\varDelta s\rightarrow 0^+$, $u_1(x)$ converges to $u_2(x)$.

##### Proof

For the standard Gaussian distribution in one dimension, the cumulative distribution function is given by $\displaystyle F(x)=\frac{1}{2}\left( 1+{{\,\mathrm{erf}\,}}\left( \frac{x}{\sqrt{2}}\right) \right)$. Thus, we obtain

8 $$\begin{aligned} {{\,\mathrm{erf}\,}}\left( \frac{x}{\sqrt{2}}\right) = 2F(x) - 1. \end{aligned}$$

By Chebyshev’s Inequality (see Lemma 1), one can conclude that for any positive *k*,

9 $$\begin{aligned} F(k)-F(-k)\geqslant 1-\frac{1}{k^2}. \end{aligned}$$

Note that $1-F(k) = F(-k)$ due to the symmetry of standard Gaussian distribution. Hence, Eq. (9) implies

$$\begin{aligned}&F(k)\geqslant 1-\frac{1}{k^2}+F(-k) = 1-\frac{1}{k^2}+1-F(k)\\ \Leftrightarrow&1-\frac{1}{k^2}\leqslant F(k)\leqslant 1, \end{aligned}$$

and analogously, $0\leqslant F(-k)\leqslant \frac{1}{k^2}$ is implied. Together with Eq. (8), it gives

$$\begin{aligned} \left\{ \begin{aligned}&1-\frac{1}{k^2}\leqslant {{\,\mathrm{erf}\,}}\left( \frac{k}{\sqrt{2}}\right) \leqslant 1,\\&-1\leqslant {{\,\mathrm{erf}\,}}\left( -\frac{k}{\sqrt{2}}\right) \leqslant -1+\frac{1}{k^2}. \end{aligned} \right. \end{aligned}$$

Let $k=\frac{s_i}{\varDelta s}>0$, for any $s_i\in (a,b)\subset (0,L), i=\{1,\dots , N_s\}$, then

$$\begin{aligned} \left\{ \begin{aligned}&1-\left( \frac{\varDelta s}{s_i}\right) ^2\leqslant {{\,\mathrm{erf}\,}}\left( \frac{s_i}{\sqrt{2}\varDelta s}\right) \leqslant 1,\\&-1\leqslant {{\,\mathrm{erf}\,}}\left( -\frac{s_i}{\sqrt{2}\varDelta s}\right) \leqslant -1+\left( \frac{\varDelta s}{s_i}\right) ^2. \end{aligned} \right. \end{aligned}$$

As it has been defined earlier that $\varDelta s = (b-a)/N_s$, we obtain

$$\begin{aligned}&\sum _{i=1}^{N_s}\left( 1-\left( \frac{\varDelta s}{s_i}\right) ^2\right) \varDelta s\leqslant \sum _{i=1}^{N_s} {{\,\mathrm{erf}\,}}\left( \frac{s_i}{\sqrt{2}\varDelta s}\right) \varDelta s\leqslant \sum _{i=1}^{N_s}\varDelta s\\ \Rightarrow&(b-a) - (\varDelta s)^3\sum _{i=1}^{N_s}\frac{1}{s_i^2}\leqslant \sum _{i=1}^{N_s} {{\,\mathrm{erf}\,}}\left( \frac{s_i}{\sqrt{2}\varDelta s}\right) \varDelta s\leqslant (b-a). \end{aligned}$$

Since $\displaystyle \lim _{\varDelta s\rightarrow 0^+}(\varDelta s)^3\sum _{i=1}^{N_s}\frac{1}{s_i^2}=0$ for any $s_i\in (a,b)\subset (0,L)$, the Squeeze Theorem (Apostol and Ablow 1958) implies that

10 $$\begin{aligned} \displaystyle \lim _{\varDelta s\rightarrow 0^+}\sum _{i=1}^{N_s}{{\,\mathrm{erf}\,}}\left( \frac{s_i}{\sqrt{2}\varDelta s}\right) \varDelta s = b-a. \end{aligned}$$

Analogously, we obtain that for any $s_i\in (a,b)\subset (0,L)$,

11 $$\begin{aligned} \displaystyle \lim _{\varDelta s\rightarrow 0^+}\sum _{i=1}^{N_s}{{\,\mathrm{erf}\,}}\left( -\frac{s_i}{\sqrt{2}\varDelta s}\right) \varDelta s = a-b. \end{aligned}$$

Thus, it can be concluded that for any series of real number $\{x_i\}\in {\mathbb {R}}^n$, when $x_{i+1}-x_i=\varDelta s$ and $x_i$ is either all positive or all negative for any $i=\{1, \cdots , N_s\}$,

12 $$\begin{aligned} \displaystyle \lim _{\varDelta s\rightarrow 0^+}\sum _{i=1}^{N_s}{{\,\mathrm{erf}\,}}\left( \frac{x_i}{\sqrt{2}\varDelta s}\right) \varDelta s = (b-a){{\,\mathrm{sgn}\,}}(x_i), \end{aligned}$$

where ${{\,\mathrm{sgn}\,}}(x)$ is the sign function defined by

$$\begin{aligned} {{\,\mathrm{sgn}\,}}(x) = \left\{ \begin{aligned}&1, \text{ if } x>0,\\&0, \text{ if } x=0,\\&-1, \text{ if } x<0.\\ \end{aligned} \right. \end{aligned}$$

We rewrite $u_1(x)$ as

$$\begin{aligned}\displaystyle u_1(x)&= P\left[ \frac{1}{2}\left( \frac{x}{L}-1\right) \sum _{i=1}^{N_s}{{\,\mathrm{erf}\,}}\left( \frac{s_i}{\sqrt{2}\varDelta s}\right) \varDelta s + \frac{1}{2}\frac{x}{L}\sum _{i=1}^{N_s}{{\,\mathrm{erf}\,}}\left( \frac{L-s_i}{\sqrt{2}\varDelta s}\right) \varDelta s \right. \\&\quad \left. - \frac{1}{2}\sum _{i=1}^{N_s}{{\,\mathrm{erf}\,}}\left( \frac{x-s_i}{\sqrt{2}\varDelta s}\right) \varDelta s \right] .\\ \end{aligned}$$

Combining Eqs. (10), (11) and (12), $u_1(x)$ is given by

$$\begin{aligned} u_1(x)&= P\left[ \frac{1}{2}\left( \frac{x}{L}-1\right) (b-a)+\frac{1}{2}\frac{x}{L}(b-a)+\frac{1}{2}[(x-b){{\,\mathrm{sgn}\,}}(x-b) \right. \\&\qquad \left. -(x-a){{\,\mathrm{sgn}\,}}(x-a)]\right] \\&= P\left[ \left( \frac{x}{L}-\frac{1}{2}\right) (b-a)+\frac{1}{2}[(x-b){{\,\mathrm{sgn}\,}}(x-b)-(x-a){{\,\mathrm{sgn}\,}}(x-a)]\right] \\&= P\left[ \left( \frac{x}{L}-\frac{1}{2}\right) (b-a)+\frac{1}{2}[|x-b|-|x-a|]\right] \\&= \left\{ \begin{aligned}&P\frac{x}{L}(b-a), \quad 0\leqslant x \leqslant a,\\&P\frac{x}{L}(b-a)-x+a, \quad a<x\leqslant b,\\&P\left( \frac{x}{L}-1\right) (b-a), \quad b<x\leqslant L,\\ \end{aligned} \right. \end{aligned}$$

Rewriting $u_2(x)$ regarding a general bounded domain gives

$$\begin{aligned} u_2(x) = \left\{ \begin{aligned}&P\frac{x}{L}(b-a), \quad 0\leqslant x \leqslant a,\\&P\frac{x}{L}(b-a)-x+a, \quad a<x\leqslant b,\\&P\left( \frac{x}{L}-1\right) (b-a), \quad b<x\leqslant L.\\ \end{aligned} \right. \end{aligned}$$

Hence, we conclude that $u_1(x)$ converges to $u_2(x)$ as $\varDelta s\rightarrow 0^+$. $\square$

#### Finite-element method solutions with arbitrary locations of biological cells

For the finite-element method, we use piecewise Lagrangian linear basis functions. We divide the computational domain into $N_e$ mesh elements, with the nodal point $x_1 = 0$ and $x_{N_e+1} = L$. For the implementation, we define the cell density as the count of biological cells in every mesh element divided by the length of the mesh element, hence, it is a constant within every mesh element and it is an interval function over the mesh elements. In other words, in the mesh element $[x_j, x_{j+1}]$, the count of the biological cell is defined by

$$\begin{aligned}N_c([x_j, x_{j+1}]) = \int _{x_j}^{x_{j+1}}n_c([x_j, x_{j+1}])dx = h n_c([x_j, x_{j+1}]),\end{aligned}$$

for any $j\in \{1,\dots ,N_e\}$, where *h* is the size of every mesh element. To clarify the notations, we use $n_c([\cdot , \cdot ])$ for the cell density function when it is an interval function in one dimension, and $n_c(x)$ when it can be written analytically as a continuous function. Same settings hold for the function of cell count $N_c$. Different from $(BVP^1_{SP})$ where $\varDelta s$ is the variance of $\delta _{\varepsilon }$, for finite-element methods, we set $\varepsilon \leqslant h/3$, such that the integration of $\delta _{\varepsilon }(x-x')$ for any $0<x'<L$ over any mesh element with size *h*, is close to 1 (see Lemma 3, which follows later). With the two approaches, the boundary value problems with Dirichlet boundary condition are defined by

13 $$\begin{aligned} (BVP^2_{SP})\left\{ \begin{aligned} -\frac{d^2u_1}{dx^2}&=P\sum _{i=1}^{N_s}\delta '_{\varepsilon }(x-s_i), x\in (0,L),\\ u_1(0)&=u_1(L)=0, \end{aligned} \right. \end{aligned}$$

and

14 $$\begin{aligned} (BVP^2_{den})\left\{ \begin{aligned} -\frac{d^2u_2}{dx^2}&= P\frac{dn_c}{dx}, x\in (0,L),\\ u_2(0)&=u_2(L)=0, \end{aligned} \right. \end{aligned}$$

where $s_i$ is the position of biological cells, and $N_s$ is the total number of cells in the computational domain. The consistency between $(BVP^2_{SP})$ and $(BVP^2_{den})$ can be verified by the following lemmas and theorem.

##### Lemma 2

Given the Gaussian distribution of mean $\mu$ and variance $\varepsilon ^2$:

$$\begin{aligned} \delta _{\varepsilon }(x) = \frac{1}{\sqrt{2 \pi \varepsilon ^2}} \exp (-\frac{(x - \mu )^2}{2 \varepsilon ^2}), \end{aligned}$$

then for any $R > 0$, it follows that

$$\begin{aligned} \lim _{\varepsilon \rightarrow 0} \int _{\mu -R}^{\mu +R} \delta _{\varepsilon }(x) d x = 1. \end{aligned}$$

##### Proof

We use the transformation $\displaystyle y^2 = \frac{(x - \mu )^2}{2 \varepsilon ^2}$, which transforms the bounds of the integral, and further changes the integral into

$$\begin{aligned} \displaystyle \int _{\mu -R}^{\mu +R} \delta _{\varepsilon }(x) d x = \frac{1}{\sqrt{\pi }} \int _{-\frac{R}{\sqrt{2} \varepsilon }}^{\frac{R}{\sqrt{2} \varepsilon }} e^{-y^2} dy. \end{aligned}$$

Sending $\varepsilon \longrightarrow 0$, makes the bounds tend to infinity, hence

$$\begin{aligned} \lim _{\varepsilon \longrightarrow 0} \int _{\mu -R}^{\mu +R} \delta _{\varepsilon }(x) d x = \frac{1}{\sqrt{\pi }} \int _{-\infty }^{\infty } e^{-y^2} dy = 1, \end{aligned}$$

since it is well known that

$$\begin{aligned} \int _{-\infty }^{\infty } e^{-y^2} dy = \sqrt{\pi }. \end{aligned}$$

This concludes the proof. $\square$

In order to quantify the error of $\varepsilon$ having a value differing from zero, one can use the following Empirical Rule:

##### Lemma 3

(Empirical rule (Pukelsheim (1994))) Given the Gaussian distribution of mean $\mu$ and variance $\varepsilon ^2$:

$$\begin{aligned} \delta _\varepsilon (x-\mu ) = 1/\sqrt{2\pi \varepsilon ^2}\exp \{-(x-\mu )^2/(2\varepsilon ^2)\}, \end{aligned}$$

then the following integration can be computed:

1. $$\int _{\mu -\varepsilon }^{\mu +\varepsilon }\delta _{\varepsilon }(x-\mu )dx\approx 0.6827;$$
2. $$\int _{\mu -2\varepsilon }^{\mu +2\varepsilon }\delta _{\varepsilon }(x-\mu )dx\approx 0.9545;$$
3. $$\int _{\mu -3\varepsilon }^{\mu +3\varepsilon }\delta _{\varepsilon }(x-\mu )dx\approx 0.9973.$$

##### Theorem 1

Denote $u^h_1(x)$ and $u^h_2(x)$ respectively the finite-element solution to $(BVP^2_{SP})$ and $(BVP^2_{den})$. With Lagrangian linear basis functions for the finite element method, $u^h_1(x)$ converges to $u^h_2(x)$, as $\varepsilon \rightarrow 0^+$ in the Gaussian distribution of the SP approach, regardless of the positions of biological cells.

##### Proof

We define $v^h(x) = u^h_1(x) - u^h_2(x)$, then $v^h(x)$ satisfies the following boundary value problem

15 $$\begin{aligned} (BVP^1_v)\left\{ \begin{aligned} -\frac{d^2v^h}{dx^2}&= P\left( \sum _{i=1}^{N_s}\delta '_{\varepsilon }(x-s_i)-\frac{dn_c}{dx}\right) , x\in (0,L),\\ v(0)&=v(L)=0. \end{aligned} \right. \end{aligned}$$

The corresponding Galerkin’s form reads as

$$\begin{aligned} (GF_v^1)\left\{ \begin{aligned}&\text {Find }v^h\in H_0^1((0, L))\text {, such that}\\&\int _0^L \frac{dv^h}{dx}\phi 'dx = \int _0^LP\left( \sum _{i=1}^{N_s}\delta '_{\varepsilon }(x-s_i)-\frac{dn_c}{dx}\right) \phi dx,\\&\text {for all }\phi \in H_0^1((0, L)). \end{aligned} \right. \end{aligned}$$

Using integration by parts and letting $\phi = \phi _j , j\in \{1, \dots , N_e\}$, the equation in $(GF_v^1)$ can be rewritten by

$$\begin{aligned} \int _0^L \frac{dv^h}{dx}\phi '_jdx&= \int _0^LP\left( \sum _{i=1}^{N_s}\delta '_{\varepsilon }(x-s_i)-\frac{dn_c}{dx}\right) \phi _j dx \\&= \left[ P\sum _{i=1}^{N_s}\delta _{\varepsilon }(x-s_i)\phi _j\right] ^L_0 - [Pn_c\phi _j]^L_0 \\&\quad - \int _{0}^L P\left( \sum _{i=1}^{N_s}\delta _{\varepsilon }(x-s_i) - n_c\right) \phi '_jdx \\ \text{(Boundary } \text{ condition) }&= - \int _{0}^L P\left( \sum _{i=1}^{N_s}\delta _{\varepsilon }(x-s_i) - n_c\right) \phi '_jdx \\&= P\sum _{j=1}^{N_e} \left\{ \int _{x_j}^{x_{j+1}}n_c \phi _j' dx -\int _{x_j}^{x_{j+1}}\sum _{i=1}^{N_s}\delta _{\varepsilon }(x-s_i)\phi '_j dx\right\} \\&= \frac{P}{h}\sum _{j=1}^{N_e} \left[ \int _{x_j}^{x_{j+1}} \sum _{i=1}^{N_s}\delta _{\varepsilon }(x-s_i)dx - N_c([x_j,x_{j+1}])\right] \\ (\varepsilon \rightarrow 0^+\text {, Lemma }2)&\rightarrow 0, \end{aligned}$$

since the number of biological cells in $[x_j, x_{j+1}]$ for any $j\in \{1,2, \dots , N_e\}$ can be defined as $\displaystyle N_c([x_j,x_{j+1}]) = \lim _{\varepsilon \rightarrow 0^+}\int _{x_j}^{x_{j+1}} \sum _{i=1}^{N_s}\delta _\varepsilon (x-s_i)dx$, where $N_s$ is the total number of biological cells in the computational domain. We note that if a cell center is located on a nodal point, such as $x_j$ or $x_{j+1}$, then only half of the unit counts as $\varepsilon \rightarrow 0^+$. We, further, note that Empirical Rule, Lemma 3, can be used to quantify the error for $\varepsilon$ not being identical zero. $\square$

## Mathematical models in two dimensions

### Smoothed particle approach and cell density approach

In the multi dimensional case, the Navier-Cauchy equation of conservation of momentum over the computational domain $\varOmega$ which is an isotropic and homogeneous domain, without considering inertia, is given by

16 $$\begin{aligned} -\nabla \cdot \varvec{\sigma }={\varvec{f}}. \end{aligned}$$

Since we consider a linear, homogeneous and isotropic domain, with Hooke’s Law, the stress tensor $\varvec{\sigma }$ is defined as

17 $$\begin{aligned} \varvec{\sigma } = \frac{E}{1+\nu }\left\{ \varvec{\epsilon }+{{\,\mathrm{tr}\,}}(\epsilon )\left[ \frac{\nu }{1-2\nu }\right] {\varvec{I}}\right\} , \end{aligned}$$

where *E* is the Young’s modulus of the material, $\nu$ is Poisson’s ratio and $\varvec{\epsilon }$ is the infinitesimal strain tensor:

18 $$\begin{aligned} \varvec{\epsilon } = \frac{1}{2}[\varvec{\nabla u}+(\varvec{\nabla u})^T]. \end{aligned}$$

Considering a subdomain $\varOmega _w\subset \varOmega$, where the center positions of the biological cells are located, then the SP approach and cell density approach with homogeneous Dirichlet boundary condition are derived by

19 $$\begin{aligned} (BVP^3_{SP})\left\{ \begin{aligned} -\nabla \cdot \varvec{\sigma }&= P_{SP}\sum _{i=1}^{N_s}\nabla \delta _{\varepsilon }({\varvec{x}}-\varvec{s_i}), {\varvec{x}}\in \varOmega ,\\ {\varvec{u}}({\varvec{x}})&= {\varvec{0}}, {\varvec{x}}\in \partial \varOmega , \end{aligned} \right. \end{aligned}$$

and

20 $$\begin{aligned} (BVP^3_{den})\left\{ \begin{aligned} -\nabla \cdot \varvec{\sigma }&= P_{den}\nabla \cdot (n_c{\varvec{I}}), {\varvec{x}}\in \varOmega ,\\ {\varvec{u}}({\varvec{x}})&= {\varvec{0}}, {\varvec{x}}\in \partial \varOmega , \end{aligned} \right. \end{aligned}$$

where ${\varvec{u}}$ is the unknown displacement field which is to be found.

### Consistency between two approaches in finite-element method

To prove the consistency between these two approaches, we define that for the triangular mesh element $e_k, k\in \{1,\dots ,N_e\}$, where $N_e$ is the total number of mesh elements in $\varOmega$, the density of biological cells $n_c(e_k)$ is constant within a mesh element and a function of the mesh element (hence it is a piecewise constant function), and hence the count of biological cells $N_c(e_k)$ is expressed by

$$\begin{aligned} N_c(e_k) = \int _{e_k}n_c(e_k)d\varOmega = A(e_k)n_c(e_k), \end{aligned}$$

where $A(e_k)$ is the area of mesh element $e_k$. To indicate that the Gaussian distribution is a proper replacement of the Dirac Delta distribution as $\varepsilon \rightarrow 0$ in two dimensions, we state the following lemma:

#### Lemma 4

Given the bivariate Gaussian distribution centered at mean $\varvec{x'}$ with variance $\varepsilon ^2$:

$$\begin{aligned} \displaystyle \delta _{\varepsilon }({\varvec{x}},\varvec{x'}) = \frac{1}{2 \pi \varepsilon ^2} \exp (-\frac{|| {\varvec{x}} - \varvec{x'} ||^2}{2 \varepsilon ^2}), \end{aligned}$$

then for any ball centered at $\varvec{x'}$ with radius *R*, denoted by ${\mathcal {B}}_R(\varvec{x'})$, it follows that

$$\begin{aligned}\displaystyle \int _{{\mathcal {B}}_R(\varvec{x'})} \delta _{\varepsilon }({\varvec{x}},\varvec{x'}) d \varOmega = 1 - e^{-\frac{R^2}{2 \varepsilon ^2}}. \end{aligned}$$

Hence, for any $R > 0$, it follows that

$$\begin{aligned} \displaystyle \lim _{\varepsilon \rightarrow 0} \int _{{\mathcal {B}}_R(\varvec{x'})} \delta _{\varepsilon }({\varvec{x}},\varvec{x'}) d \varOmega = 1. \end{aligned}$$

#### Proof

We use centered polar coordinates, $x = x' + r \cos (\theta )$ and $y = y' + r \sin (\theta )$, and the Jacobian of the transformation, to arrive at

$$\begin{aligned}\displaystyle \int _{{\mathcal {B}}_R(\varvec{x'})} \delta _{\varepsilon }({\varvec{x}},\varvec{x'}) d \varOmega = \int _0^{2 \pi } \int _0^R \frac{1}{2 \pi \varepsilon ^2} \exp (-\frac{r^2}{2 \varepsilon ^2}) r d r d \theta = 1 - e^{-\frac{R^2}{2 \varepsilon ^2}}. \end{aligned}$$

Treating $R > 0$ as an arbitrary constant, and sending $\varepsilon$ to zero, gives

$$\begin{aligned} \displaystyle \lim _{\varepsilon \rightarrow 0} \int _{{\mathcal {B}}_R(\varvec{x'})} \delta _{\varepsilon }({\varvec{x}},\varvec{x'}) d \varOmega = 1. \end{aligned}$$

This concludes the proof. $\square$

Then, similar to the one dimensional case, we state the following theorem:

#### Theorem 2

Let $\varvec{u^h_1}({\varvec{x}})$ and $\varvec{u^h_2}({\varvec{x}})$, respectively, denote the finite-element solution to $(BVP^3_{SP})$ and $(BVP^3_{den})$ with $P_{SP}=P_{den}=P$. With Lagrangian linear basis functions for the finite element method, $\varvec{u^h_1}({\varvec{x}})$ converges to $\varvec{u^h_2}({\varvec{x}})$, as $\varepsilon \rightarrow 0^+$ in the Gaussian distribution of the SP approach, regardless of the positions of biological cells.

#### Proof

We consider $\varvec{v^h}({\varvec{x}}) = \varvec{u^h_1}({\varvec{x}}) - \varvec{u^h_2}({\varvec{x}})$, $\varvec{v^h}({\varvec{x}})$ satisfies the following boundary value problem:

21 $$\begin{aligned} (BVP^3_v)\left\{ \begin{aligned} -\nabla \cdot \varvec{\sigma }&= P\left[ \sum _{i=1}^{N_s}\nabla \delta _{\varepsilon }({\varvec{x}}-\varvec{s_i}) - \nabla \cdot (n_c{\varvec{I}})\right] , {\varvec{x}}\in \varOmega ,\\ {\varvec{v}}({\varvec{x}})&= {\varvec{0}}, {\varvec{x}}\in \partial \varOmega . \end{aligned} \right. \end{aligned}$$

The corresponding Galerkin’s form reads as

$$\begin{aligned} (GF^3_v)\left\{ \begin{aligned}&\text {Find }\varvec{v^h}\in \varvec{H_0^1}(\varOmega )\text {, such that}\\&\int _\varOmega \varvec{\sigma }(\varvec{v^h}):\nabla \varvec{\phi ^h} d\varOmega = \int _\varOmega P\left[ \sum _{i=1}^{N_s}\nabla \delta _{\varepsilon }({\varvec{x}}-\varvec{s_i}) - \nabla \cdot (n_c{\varvec{I}})\right] \cdot \varvec{\phi ^h} d\varOmega ,\\&\text {for all }\varvec{\phi ^h}\in \varvec{H_0^1}(\varOmega ). \end{aligned} \right. \end{aligned}$$

With integration by parts and letting $\varvec{\phi ^h} = \varvec{\phi ^h_k} , k\in \{1, \dots , N_e\}$, the equation in $(GF_v^3)$ can be rewritten by

$$\begin{aligned} \int _\varOmega \varvec{\sigma }(\varvec{v^h}):\nabla \varvec{\phi ^h_k} d\varOmega&= \int _\varOmega P\left[ \sum _{i=1}^{N_s}\nabla \delta _{\varepsilon }({\varvec{x}}-\varvec{s_i}) - \nabla \cdot (n_c{\varvec{I}})\right] \cdot \varvec{\phi ^h_k} d\varOmega \\&= P\left\{ \left[ \int _{\partial \varOmega } \sum _{i=1}^{N_s}\delta _{\varepsilon }({\varvec{x}}-\varvec{s_i})\varvec{\phi ^h_k}\cdot {\varvec{n}}d\varGamma -\int _\varOmega \sum _{i=1}^{N_s}\delta _{\varepsilon }({\varvec{x}}-\varvec{s_i})\nabla \cdot \varvec{\phi ^h_k}d\varOmega \right] \right. \\&\quad \left. -\left[ \int _{\partial \varOmega }n_c\varvec{\phi ^h_k}\cdot {\varvec{n}}d\varGamma - \int _\varOmega n_c\nabla \cdot \varvec{\phi ^h_k}d\varOmega \right] \right\} \\ \text{(Boundary } \text{ condition) }&= -P\int _\varOmega \sum _{i=1}^{N_s}\delta _{\varepsilon }({\varvec{x}}-\varvec{s_i})\nabla \cdot \varvec{\phi ^h_k} - n_c\nabla \cdot \varvec{\phi ^h_k}d\varOmega \\&= - P\sum _{k=1}^{N_e}\int _{e_k} \sum _{i=1}^{N_s}\delta _{\varepsilon }({\varvec{x}}-\varvec{s_i})\nabla \cdot \varvec{\phi ^h_k} - n_c\nabla \cdot \varvec{\phi ^h_k}d\varOmega \\&= -P\sum _{k=1}^{N_e}\nabla \cdot \varvec{\phi ^h_k}\int _{e_k} \sum _{i=1}^{N_s}\delta _{\varepsilon }({\varvec{x}}-\varvec{s_i}) - n_c d\varOmega \\&= P\sum _{k=1}^{N_e}\nabla \cdot \varvec{\phi ^h_k}\left[ N_c(e_k) - \int _{e_k} \sum _{i=1}^{N_s}\delta _{\varepsilon }({\varvec{x}}-\varvec{s_i})d\varOmega \right] \\ (\varepsilon \rightarrow 0^+\text {, Lemma }4)&\rightarrow 0, \end{aligned}$$

since it can be defined that $\displaystyle N_c(e_k) = \lim _{\varepsilon \rightarrow 0^+}\int _{e_k} \sum _{i=1}^{N_s}\delta _\varepsilon ({\varvec{x}}-\varvec{s_i})d\varOmega$, where $N_s$ is the total number of biological cells in the computational domain. Note that in two dimensions, $\varepsilon$ needs to be sufficiently small compared to the size of a triangular mesh element. Korn’s inequality (Braess 2007) and symmetry (boundedness) conclude the theorem. We note that if cell is located on a face of an element, then its contribution only counts for a half as $\varepsilon \rightarrow 0$ . If a cell is located at an element vertex, then its contribution only counts for the angle of the vertex in the current element divided by $2 \pi$ as $\varepsilon \rightarrow 0$. $\square$

We note that Lemma 4 can be used to estimate the error when one wishes to accommodate for $\varepsilon$ not being identically zero.

## Simulation results

Simulation results in both one and two dimensions are discussed in this section. Since the objective of this manuscript is to investigate the consistency and the connections between the SP approach and the cell density approach, all the parameters are dimensionless. For one and two dimensional simulations, the parameter values are shown in Tables 1 and 5, respectively.

### One-dimensional results

**Fig. 2:**
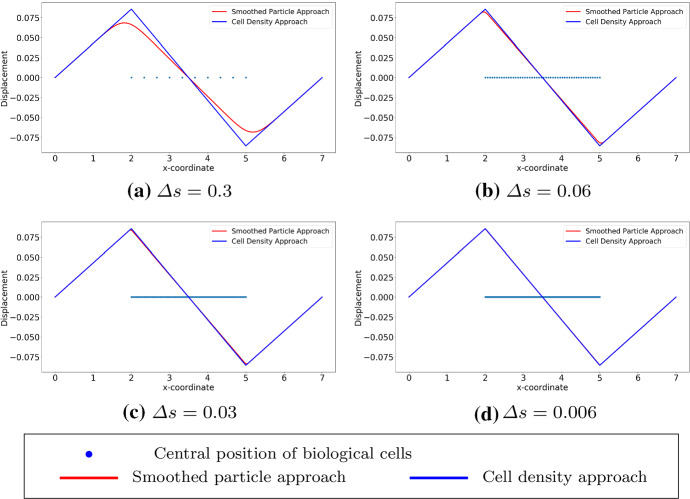
The exact solutions to $(BVP^1_{SP})$ and $(BVP^1_{den})$ are shown, with various values of $\varDelta s$, which is the distance between centre positions of any two adjacent biological cells. Blue points are the centre positions of biological cells. Red curves represent the solutions to $(BVP^1_{SP})$ and blue curves represent the solutions to $(BVP^1_{den})$

We show the results by analytical solutions (see Eqs. (5) and (7) for the SP approach and the cell density approach, respectively) in Fig. 2 with various values of $\varDelta s$ (i.e. depending on different number of biological cells in the subdomain (*a*, *b*)). Here, the computational domain is (0, 7) with $L=7$ and the subdomain where the biological cells locate uniformly is (2, 5) with $a=2$ and $b=5$. With the decrease of the variance in the Gaussian distribution in $(BVP^2_{SP})$, the curves gradually overlap, which verifies the convergence between the analytical solutions to these two approaches.

**Table 1:** Model parameter values that are used in one dimensional simulations

Parameter	Value	Description
*E*	1	Stiffness of the computational domain (i.e. ECM)
*P*	0.01	Force magnitude in both approaches as indicated in $(BVP_v^1)$ in Theorem 1
*L*	7	The right endpoint of the computational domain
*a*	2	The left endpoint of the subdomain (i.e. the wound region)
*b*	5	The right endpoint of the subdomain (i.e. the wound region)

**Fig. 3:**
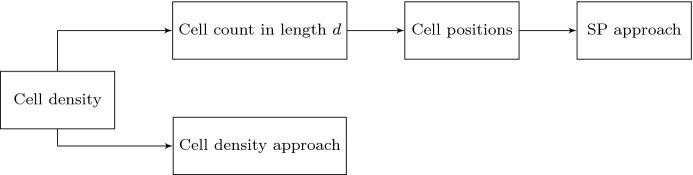
With exact expression of cell density function and the first order derivative of the function exists, cell density approach is implemented directly. Based on the cell density, the number of cells in a certain region with length *d* is determined and subsequently, the center positions of cells can be generalized. Hence, the SP approach is implemented

**Fig. 4:**
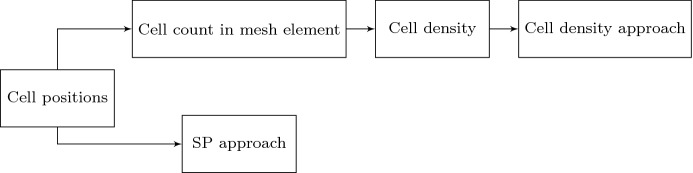
Given the center positions of cells, one can directly implement the SP model. Computing the number of cells in every mesh element and divided by the length of the mesh element results into the cell density. Subsequently, cell density approach can be implemented

To implement the model, there are two different algorithms shown in Figs. 3 and 4. Depending on different circumstances, the implementation method is elected. The cell density in one dimension is defined as the number of cells per length unit. In other words, the cell count in a given domain can be computed by integrating the cell density over the domain. If the cell density function can be expressed analytically and the first order derivative of the function exists, then a certain bin length *d* is chosen and the cell count in every bin of *d* length is calculated. Then we generalize the center positions of cells in every bin of length *d*, thus, the SP approach can be implemented, as it is indicated in Fig. 3. However, it is not always straightforward to obtain the analytical expression of cell density. If the center positions of cells are given, then the SP approach can be implemented directly, and the number of cells in each mesh element can be counted. Hence, the cell density will be computed analogously at each mesh points, as it is shown in Fig. 4. Therefore, the boundary value problem of cell density approach is solved by numerical methods, for example, the finite-element methods. In summary, in Fig. 3 when the cell density is prescribed and on the basis of this cell density function, we assign a number of cells in each line element (in one dimension) or triangular (in two dimensions) element. This amounts to using a constant cell density in each element (line element or triangular element). The approach shown in Fig. 4 is the contrary, where cell density cannot be expressed analytically and then the number of cells in each element is counted and divided by the correspond measure (length in one dimension and element area in two dimensions) to reconstruct the cell density distribution. In both cases, the cell density is piecewise constant over the domain of computation in which the cell density is constant over each element.

**Fig. 5:**
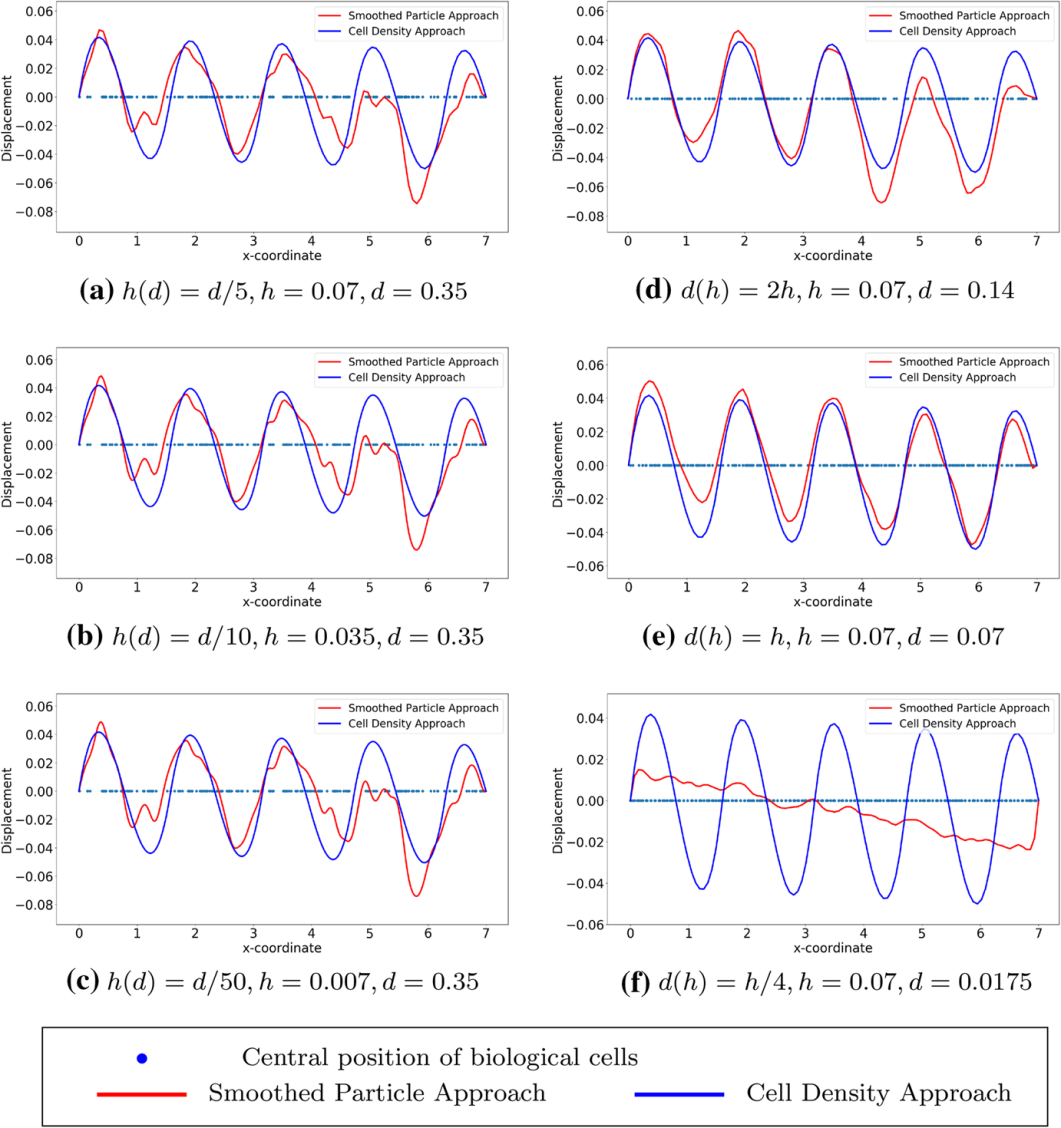
The cell density function is sine function and using the algorithm in Fig. 3, different simulations are carried out with various mesh size and the total number of cells. Blue curves represent the solutions to $(BVP^2_{SP})$, and red curves are the solutions to $(BVP^2_{den})$ with $n_c(x) = 40|\sin (2x)|$. In (**a**)–(**c**), we set $d = 0.35$ and cell positions are fixed. From (**d**) to (**f**), we use the same finite-element method settings (where *h* is efficiently small with $h = 0.07$), and we take different values of *d*

**Fig. 6:**
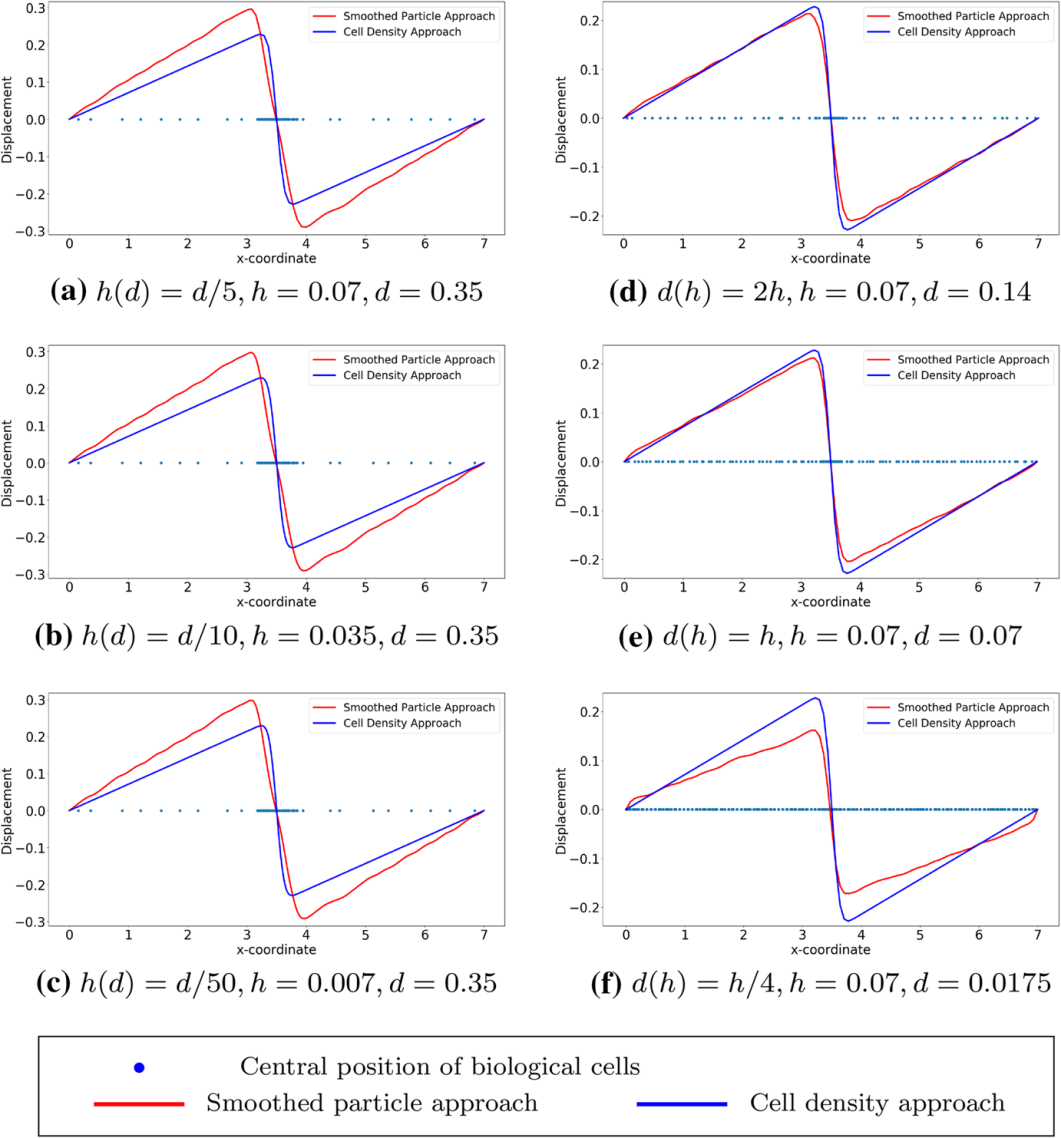
The cell density function is Gaussian distribution and using the algorithm in Fig. 3, different simulations are carried out with various mesh size and the total number of cells. Blue curves represent the solutions to $(BVP^2_{SP})$, and red curves are the solutions to $(BVP^2_{den})$ with $n_c(x) = 50\times 1/\sqrt{2\pi \times 0.1^2}\exp \{-(x-3.5)^2/(2\times 0.1^2)\}$. In (**a**)–(**c**), we set $d = 0.35$ and cell positions are fixed, as *h* is decreasing. From (**d**) to (**f**), we use the same finite-element method settings (where *h* is sufficiently small with $h = 0.07$), and simulations are carried out with various values of *d*

In this manuscript, all the numerical results have been obtained by finite-element methods with Lagrangian linear basis functions. Regarding the first implementation method (see Fig. 3), we show the results with a sine function and Gaussian distribution as cell density functions; see Figs. 5 and 6 , respectively. We start with the simulations in which we keep the number of cells and the center positions of the cells the same, then we refine the mesh. In Fig. 5(a)–(c), the bin length *d* is 0.35, and the mesh size is a function of *d*. The results that were obtained using the SP approach become smoother. With various values of *d*, the solutions to the approaches are overlapping only when the factor between the *d* and mesh size is closer to 1. From Fig. 5(d) to (f), the mesh is fixed and we vary the value of *d*. We note that in Fig. 5(f), the solution to the SP approach is significantly different from the solution to the cell density approach. This difference is mainly caused by the fact that *d* is too small and there is barely any fluctuation with the count of cells in every subdomain with length *d*, while with the Gaussian distribution as the cell density function, the majority of the cells are centered around $x=3.5$. Hence, the solution that was obtained from the SP approach still manages to be comparable with the solution to the cell density approach; see Fig. 6(f). Numerical results of the simulation in Fig. 6 are displayed in Table 2. There are some noticeable differences between two approaches, in particular the convergence rate in the $H^1$-norm: thanks to the given, differentiable cell density function, the cell density approach converges faster. In addition, the cell density approach requires less computational time with a factor of 15.

**Table 2:** Numerical results of two approaches in one dimension, where the cell density function is Gaussian distribution: $n_c(x) = 50\times 1/\sqrt{2\pi \times 0.1^2}\exp \{-(x-3.5)^2/(2\times 0.1^2)\}$. Here, we define $N_s=88$ and the mesh size $h = 0.07$. The results are solved by finite-element method with algorithm in Fig. 3

	SP Approach	Cell Density Approach
$L^2$-**norm of the solution** *u*		
$\Vert u\Vert _{L^2((0,L))}^{2h}$	0.5419891028287082	0.3581360438718032
$\Vert u\Vert _{L^2((0,L))}^h$	0.5441481069175041	0.36197930815501245
$\Vert u\Vert _{L^2((0,L))}^{h/2}$	0.5448668763153627	0.3630629995429662
Convergence rate of $L^2$-norm	1.75281178	1.826378221
$\Vert u\Vert _{H^1(((0,L))}^h$	0.9642151731656272	0.871720645462775
Reduction ratio of the subdomain (*a*, *b*) (%)	13.88062	9.52381
Relative ratio of the subdomain (*a*, *b*) after deformation (%)	86.11938	90.47619
Time cost (*s*)	0.045070	0.0032084

**Fig. 7:**
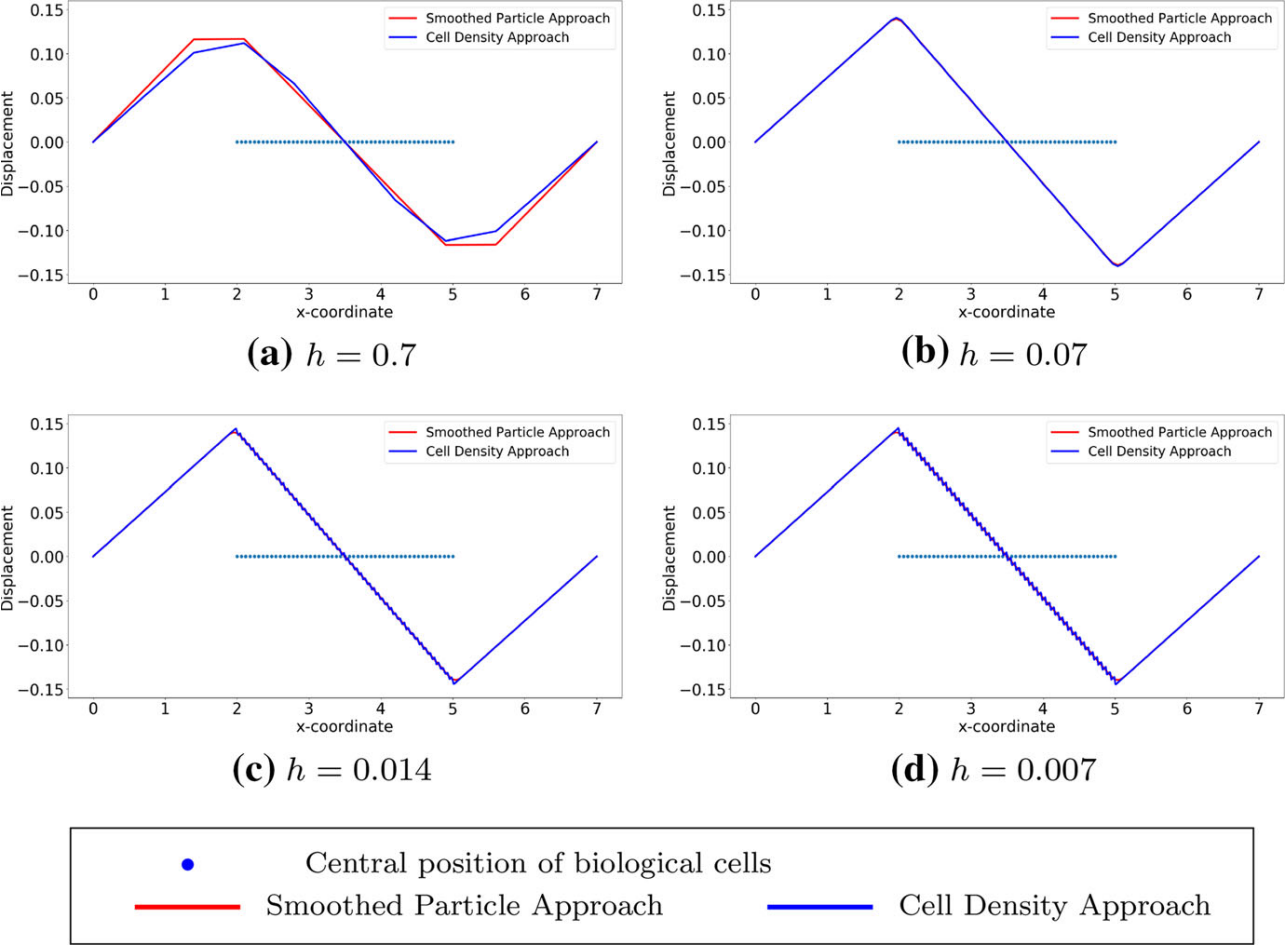
The finite-element method solutions to $(BVP^2_{SP})$ and $(BVP^2_{den})$ are shown where cells are uniformly located. With the fixed positions of cells, the solutions are convergent as $h \rightarrow 0^+$. Blue points are the centre positions of biological cells. Red curves represent the solutions to $(BVP^2_{SP})$ and blue curves represent the solutions to $(BVP^2_{den})$

**Fig. 8:**
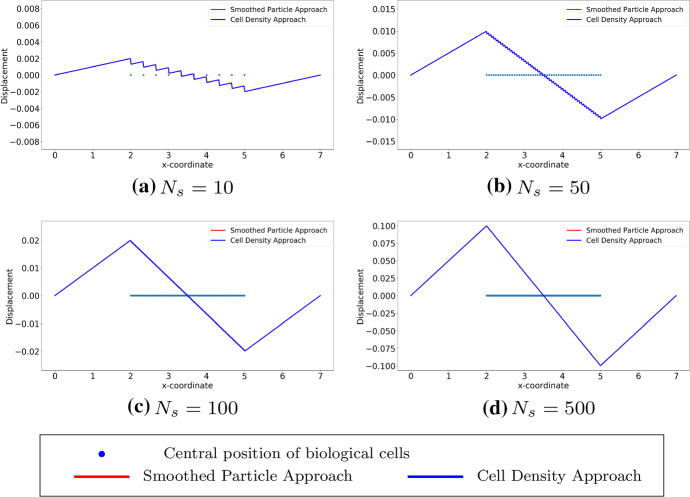
The finite-element method solutions to $(BVP^2_{SP})$ and $(BVP^2_{den})$ are shown with uniform distribution. Compared to the analytical result, the consistency between two approaches are unrelated to the number of cells, and the solutions are convergent as $h \rightarrow 0^+$. Here, we use $h = 0.007$. Blue points are the centre positions of biological cells. Red curves represent the solutions to $(BVP^2_{SP})$ and blue curves represent the solutions to $(BVP^2_{den})$

We consider cells that are located uniformly in the subdomain (2, 5), which implies that the derivative of the cell density vanishes inside the subdomain but does not exist at two endpoints of the subdomain. Since the exact solutions of both approaches are known as $u_1(x)$ in Eq. (5) and $u_2(x)$ in Eq. (7), respectively, one can perform root-mean-square error (RMS) analysis, which is given by

$$\begin{aligned} \displaystyle error = \sqrt{\frac{\sum _{i=1}^{N}[u^{exact}(x_i) - u^h(x_i)]^2}{N}}, \end{aligned}$$

where *N* is the number of mesh nodal points and $x_i$ is the coordinate of $i-$th mesh nodal point. Note that the error above is actually $L^2-$ error computed in the computational domain. To obtain the numerical results and since the cell density can be written analytically, we utilize the implementation method in Fig. 4, as the center positions of the cells are given, then the local cell density can be calculated per unit area. Compared with the results shown in Fig. 2, the results in Figs. 7 and 8 show the solutions to $(BVP^2_{SP})$ and $(BVP^2_{den})$ respectively. Note that, in the finite-element method solutions, the magnitude of the forces in both approaches are the same, and the variance of $\delta _{\varepsilon }(x)$ is related to *h* rather than $\varDelta s$. Furthermore, these figures verify that the convergence between SP approach and cell density approach is determined by the mesh size rather than by the distance between any two adjacent cells. Table 3 displays the numerical results of the simulation in Fig. 7, the reduction ratio of the subdomain and the computational cost. Similarly to the figures, there is no significant difference between the norms and the deformed length of the subdomain. However, the simulation time in the cell density approach is much shorter than in the SP approach with a factor of 35.

### Two-dimensional results

In the multi-dimensional case, we are not able to write the analytical solution to the boundary value problems. The results are all solved by the use of the finite-element method applied to $(BVP^3_{SP})$ and $(BVP^3_{den})$. Note that the force magnitude of both boundary value problems is the same. Following the same implementation methods as in one dimension, simulations are carried out with two formulas of cell density: (1) the cell density function is in the form of the standard Gaussian distribution over the computational domain with $n_c({\varvec{x}}) = 50\times \frac{1}{2\pi }\exp \left\{ -\frac{\Vert {\varvec{x}}|^2}{2}\right\}$; (2) cells are located inside the subdomain $\varOmega _w$ randomly by the uniform distribution. Implementation methods in Figs. 3 and 4 are applied respectively in Simulation (1) and (2).

**Table 3:** Numerical results of two approaches in one dimension with biological cells located uniformly. Here, we define mesh size $h = 0.07$ and $N_s = 50$, which means $\varDelta s = 0.06$. The results are solved by finite-element method with algorithm in Fig. 4

	SP Approach	Cell Density Approach
RMS error	$6.70984672\times 10^{-7}$	0.010295901
$L^2$-**norm of the solution** *u*		
$\Vert u^{h}\Vert _{L^2((0,L))}$	0.21470072397236404	0.2190152169099139
$\Vert u^{h/2}\Vert _{L^2((0,L))}$	0.2180816688838546	0.2192106132836521
$\Vert u^{h/4}\Vert _{L^2((0,L))}$	0.21897037944459403	0.219296655339645
Convergence rate of $L^2-norm$	1.927640972	1.831863826
Reduction ratio of the subdomain (*a*, *b*) (%)	7.96908	7.98821
Relative ratio of the subdomain (*a*, *b*) after deformation (%)	92.03092	92.01179
Time cost (*s*)	0.10391	0.0030458

According to the setting of the simulation, we define the cell density function by

$$\begin{aligned} n_c({\varvec{x}}) = 50\times \frac{1}{2\pi }\exp \left\{ -\frac{\Vert {\varvec{x}}|^2}{2}\right\} , \hbox { in}\ \varOmega , \end{aligned}$$

which is a Gaussian distribution multiplied by a positive constant. Similarly, in two dimensions, the cell density is defined by the number of biological cells per unit area. In other words, the cell count is computed by the local cell density multiplied by the area of selected region. Here, we assume that the selected region is a $1\times 1$ square, then we generate the center positions of biological cells in every unit square based on the local number of cells.

**Fig. 9:**
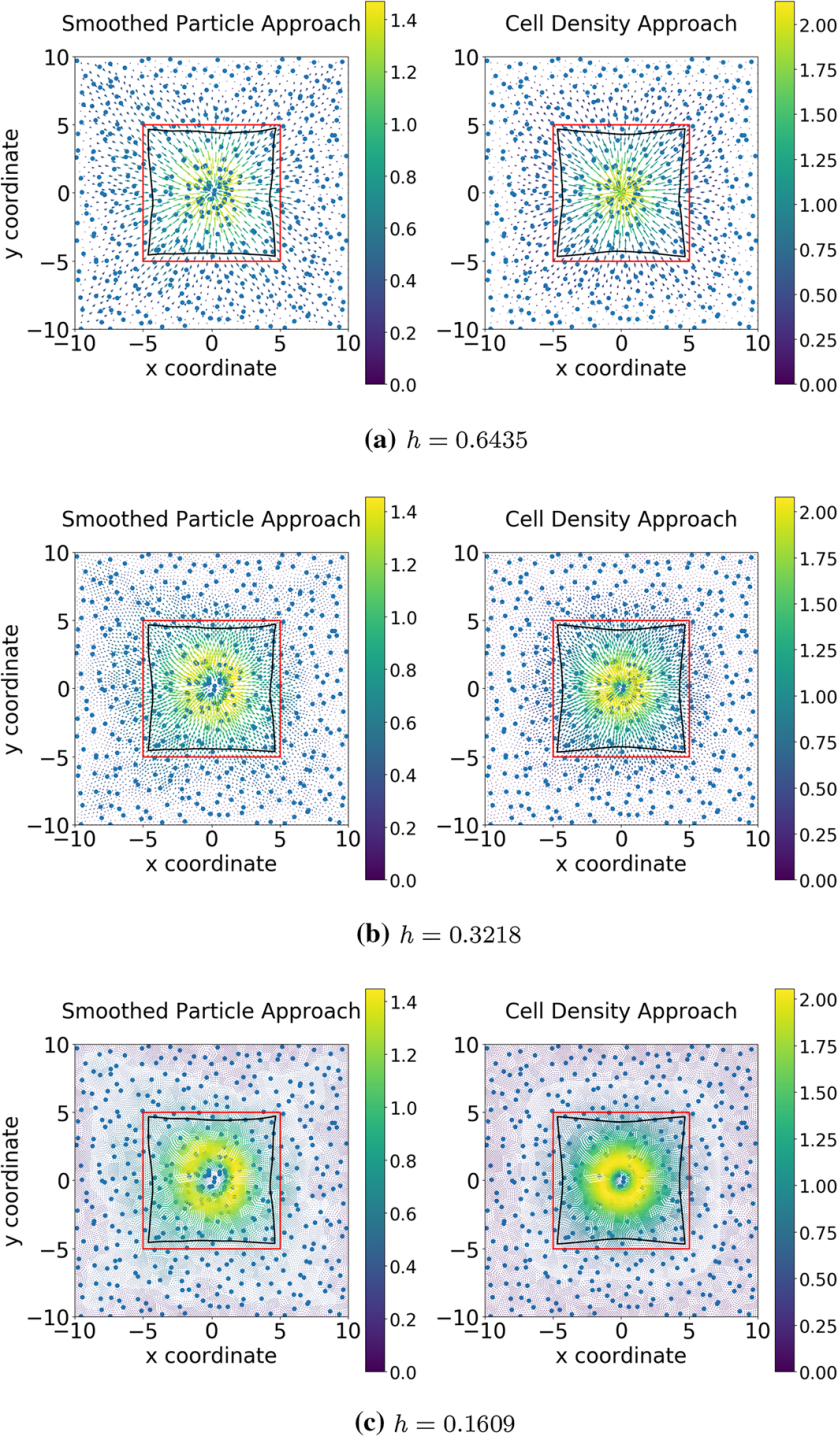
The displacement results are shown, which are solved from $(BVP^3_{SP})$ and $(BVP^3_{den})$ when cells are located according to the cell density function $n_c({\varvec{x}}) = 50\times \frac{1}{2\pi }\exp \{-\frac{\Vert {\varvec{x}}|^2}{2}\}$. Hence, implementing algorithm in Fig. 4 is used. There are 440 biological cells in the computational domain. Blue points are the center positions of biological cells, red curves are the original shapes of the subdomain, and the black curves represent the deformed boundary of the subdomain

In the two-dimensional calculations (see Fig. 9), we consider a square domain for the tissue. Within this domain, there is an artificial scar, which is also square-shaped in the current simulations, and which is indicated by the red line segments. The scar, which is bounded by the red line segments, is populated with cells (fibroblasts) that exert pulling forces. The cells are positioned randomly in the scar region such that they do not overlap. In the smoothed particle approach, the gradient of the mollified Dirac delta distribution, which amounts to a Gaussian distribution, is used to model the force exerted by each cell, whereas in the cell density approach a cell density field is reconstructed from the randomized cell positions using the procedure outlined in $(BVP_{SP}^3)$ and $(BVP_{den}^3)$; see Sect. 3.1. Summarized, in both cases in Fig. 9, the forces result from cells that are located in the square-shaped scar region that is enclosed by the red line segments.

The pulling forces that are exerted by the cells cause a displacement field over the entire domain of computation (the entire tissue region). The red line segments, which indicate the boundary between scar (containing the cells) and undamaged tissue (not containing cells that exert pulling forces), are displaced as a result of the displacement field caused by the exertion of pulling forces by the cells. The displaced interface between the damaged tissue and scar region is indicated by the black curve enclosed by the red line segments. Since on an average, the cell position is approximately in the center of the domain, the displacements are, on an average, directed towards the center. Furthermore, since the displacement is set to zero at the boundaries of the domain of computation, the magnitude of the displacement vector decreases away from the center. Since the distance between the center and the positions on the red line segments is minimal at the midpoints and maximal at the corners of the red line segments. This causes the contraction and the curved shape of the edge between the scar and undamaged region (the black curve within the red square). In both cases, the finite element solution is in $H^1(\varOmega ) \cap C^0(\varOmega )$ due to the mollified force expressions (gradient of Gaussian distribution), which do not admit any jump discontinuities of the displacement field, and as a result of the use of $C^0$ Lagrangian elements. If the forces exerted by the cells cease, then the boundary between the scar and undamaged region retract back to the red line segments.

Fig. 9 shows the numerical results regarding two approaches. There is no significant difference if the same mesh resolution is used. As the mesh is refined, the solution to the SP approach is smoother, since the “ring” in the center becomes more dominant. In Table 4, it can be concluded that there are no significant differences between the two approaches except for the computational efficiency and the convergence rate of $H^1$-norm. If the mesh is not fine enough, then the solution to the SP approach is less smooth, hence, the determination of the gradient of the solution is less accurate.

**Table 4:** Numerical results of two approaches in two dimensions with Gaussian distribution for the positions of biological cells. Figure 3 is implemented and there are 440 biological cells in the computational domain

	SP Approach	Cell Density Approach
$L^2$-**norm of the solution** *u*		
$\Vert u^h\Vert _{L^2((0,L))}$	11.14304909846569	13.188441094735877
$\Vert u^{h/2}\Vert _{L^2((0,L))}$	11.180094316889074	13.248171247805358
$\Vert u^{h/4}\Vert _{L^2((0,L))}$	11.19130107517498	13.264669660152608
Convergence rate of $L^2-norm$	1.724918322	1.856132219
$\Vert u\Vert _{H^1((0,L))}^h$	12.77795210802095	15.533928099123479
Reduction ratio of the subdomain $\varOmega _w$ (%)	19.65854	20.55949
Relative ratio of the subdomain $\varOmega _w$ after deformation (%)	80.34146	79.44051
Time cost (*s*)	0.62347	0.017315

**Table 5:** Estimated parameter values that are used in one dimensional simulations

Parameter	Value	Description
*E*	1	Stiffness of the computational domain (i.e. ECM)
*P*	10	Force magnitude in both approaches as indicated in $(BVP_v^3)$ in Theorem 2
$\nu$	0.49	Poisson’s ratio of the computational domain (i.e. ECM)
$x_0$	20	The length of the computational domain in x-coordinate
$y_0$	20	The length of the computational domain in y-coordinate
*wx*	10	The length of the subdomain (i.e. the wound region) in x-coordinate
*wy*	10	The length of the subdomain (i.e. the wound region) in y-coordinate

**Fig. 10:**
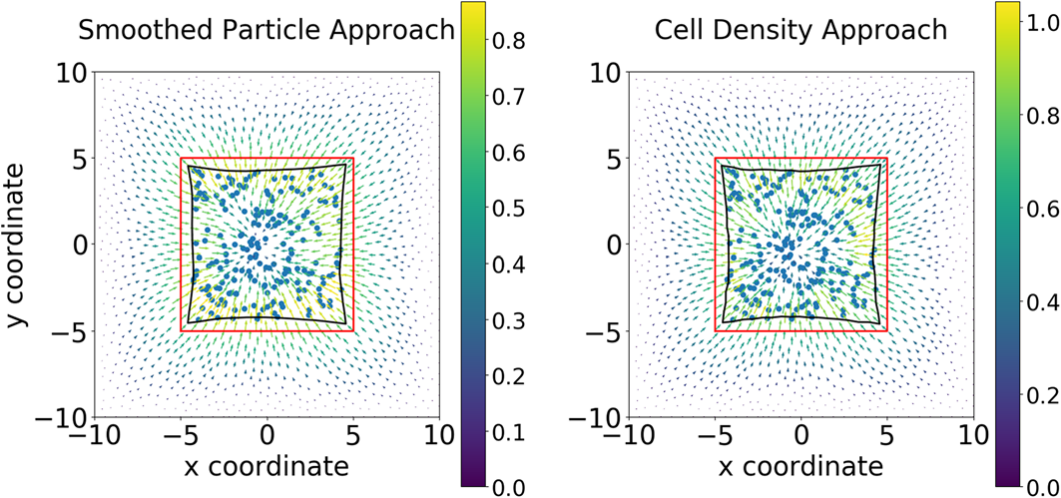
The displacement results are shown, which are solved from $(BVP^3_{SP})$ and $(BVP^3_{den})$ when cells are randomly located in the subdomain $(-5,5)\times (-5,5)$. In other words, it is impossible to write the analytical expression of $n_c({\varvec{x}})$, subsequently, the algorithm in Fig. 4 is selected. There are 196 biological cells in the computational domain. Blue points are the center positions of biological cells, red curves are the original shapes of the subdomain, and the black curves represent the deformed boundary of the subdomain

For Simulation (2), no analytical expression for (the derivative of) the density function is available. Therefore, the implementation starts with generating the cell positions, according to the principles outlined in Fig. 4. In Fig. 10, the displacement results are displayed. From the figures, hardly any significant differences between the solutions can be observed, which indicates that these two approaches are numerically consistent. Table 6 displays more details about the two approaches regarding the numerical analysis: most data are more or less the same. However, as it has been mentioned earlier, the agent-based model is computationally more expensive than the continuum-based model; here, the difference is a factor of 240.

## Conclusions

In this manuscript, we discussed the different models to simulate the pulling forces exerted by the (myo)fibroblasts depending on different scales of the wound region. We started from one dimension and later extended the models to two dimensions. In one dimension, we can write explicitly the analytical solution to the boundary value problem with specific distribution of the locations of biological cells and we proved the convergence and the consistency between the solutions to these two approaches in Sect. 2.3.1. In both one and two dimensions, the numerical solutions delivered by finite-element methods with Lagrangian linear basis functions implied that these two models are consistent under certain mesh conditions (when the mesh size is sufficiently small) and regardless the locations of the biological cells and the implementation methods. In summary, regarding the displacement of the ECM from the mechanical model, the agent-based model and the cell density model are consistent from a computational point of view. This could be used to transfer one type of model to the other one regarding the force balance in the wound healing model, as the connection between these two models has been suggested. We want to use the developed insights for the analysis of upscaling between agent-based and continuum-based model formulations.

**Table 6:** Numerical results of two approaches in two dimensions with random distribution for the positions of biological cells. Due to the nonexistence of divergence or gradient of cell density function, implementation method in Fig. 4 is used

	SP Approach	Cell Density Approach
$\Vert u^h\Vert _{L^2((0,L))}$	10.105858093727422	12.314518769308366
Reduction ratio of the subdomain $\varOmega _w$ (%)	24.24192	23.33667
Relative ratio of the subdomain $\varOmega _w$ (%)	75.75808	76.66333
Time cost (*s*)	4.20677	$1.77219\times 10^{-2}$
